# Chromatin deactivation in pregranulosa cells contributes to primordial follicle formation

**DOI:** 10.1016/j.jbc.2025.110598

**Published:** 2025-08-14

**Authors:** Xuzhao Wang, Xin Qiu, Yuxiao Ma, Xiaotong Guo, Jiahui Wei, Feiyi Wang, Heng Wang, Guiyu Zhu

**Affiliations:** College of Animal Science, Shandong Provincial Key laboratory for Livestock Germplasm Innovation & Utilization, Shandong Agricultural University, Taian, China

**Keywords:** chromatin deactivation, primordial follicle, pre-granulosa cell, chicken, oocyte

## Abstract

The establishment and maintenance of the primordial follicle (PF) pool are critical for determining the ovarian reserve in females, ensuring efficient oocyte production, particularly in farm animals such as egg-laying chickens. This process involves the breakdown of germ cell cysts and the encapsulation of individual oocytes by somatic pregranulosa cells (pre-GCs) to form PFs, which then enter a lasting state of dormancy. The proper development of pre-GCs is essential for their interaction with and protection of oocytes, facilitating successful follicle formation. How pre-GCs organize to regulate PF formation and thesubsequent quiescence entry remain poorly understood. This study investigated gene expression and chromatin regulation in chicken pre-GCs during the PF establishment to identify epigenetic mechanisms that could enhance ovarian reserve and improve egg production. Cleavage Under Targets and Tagmentation sequencing assay revealed a global reduction in active chromatin as evidenced by the depletion of acetylation of lysine 27 on histone H3 deposition. This chromatin deactivation caused the downregulation of target genes involved in promoting cell apoptosis and blocking cell adhesion, thereby supporting cell survival and enhancing cell–cell communications necessary for follicle assembly. Further validation using an *ex vivo* chicken ovary organ culture system demonstrated that prolonged chromatin activation, achieved through histone deacetylase inhibitor–mediated chromatin acetylation, significantly reduced the number of PFs and resulted in the persistence of unruptured germ cell cysts. Forcibly deactivating the chromatin increased PF formation. Therefore, the chromatin deactivation regulates the establishment of PFs and their entry into quiescent states, offering potential strategies to improve ovarian reserve and egg production in different animals.

Primordial follicles (PFs), the fundamental reproductive units of the ovary, determine the reproductive capacity of females. The formation of PFs marks the onset of folliculogenesis and establishes the foundation for future egg production. PFs are multicellular structures comprising a single oocyte surrounded by supporting pre-granulosa cells (pre-GCs). In mammals, such as mice, the development of oocytes and GCs begins in the fetal ovaries during midgestation. Female primordial germ cells (PGCs) migrate to the genital ridge, proliferate through mitosis, and form clusters known as germline cysts or nests (1). During this process, germ cells initiate meiosis and differentiate into oocytes, arresting at the diplotene stage. Simultaneously, pre-GCs are recruited from follicle-supporting progenitor cells in the ovarian surface epithelium to encircle the oocytes and form PFs. A key feature of follicle assembly is the extensive loss of oocytes through programmed cell death, with only about one-third of oocytes surviving to become PFs (2). The successful formation of PFs relies on the synchronized development and interaction between oocytes and ovarian somatic cells. Oocytes must enter and arrest at the diplotene stage of the first meiotic prophase, and there must be an adequate number of competent pre-GCs for efficient follicle assembly.

In chickens, female PGCs are initially formed in the germinal crescent, and they migrate to the gonads through the vascular system, which is a unique feature of germ cell development in birds. Upon arrival in the gonads, chicken PGCs undergo mitosis and initiate meiosis in a manner similar to mammals. However, the development of somatic pre-GCs in chicken ovaries follows distinct developmental trajectories compared with those in mammals. Unlike in mammals, where the ovarian surface epithelium gives rise to supporting cell lineages, such as Sertoli or pre-GCs, chicken embryos generate a nonsteroid-producing stromal cell population instead (3). In chickens, supporting cells originate from the mesenchyme during early gonadal development (3, 4). These mesenchymal-derived cells exhibit distinct molecular signatures, including the expression of transcription factors, such as PAX2, DMRT1, and OSR1, as well as the signaling molecule WNT4. In contrast, in mice, supporting cells arise from a population of GATA4-positive cells in the fetal ovarian cortex that initially express the leucine-rich repeat–containing G-protein–coupled receptor family gene, Lgr5 (5, 6). These Lgr5-positive cells in the cortical region are thought to be precursors to pre-GCs, which eventually form PFs. These cells encapsulate germ cells within the cortical niche of embryonic ovaries and exhibit dynamic alterations in gene expression during the neonatal period (7, 8). Despite these insights, the mechanisms by which pre-GCs locate, interact with, and encapsulate germ cells, as well as the intrinsic molecular pathways directing their behavior during PF formation, remain poorly understood.

Chromatin activation is a key epigenetic mechanism that regulates gene expression and cellular function. Histone acetylation, in particular, plays a vital role in modulating chromatin structure and transcriptional activity. Typically, histone acetylation facilitates the dissociation of DNA from the histone octamer, leading to a relaxed nucleosomal structure. This relaxation enables transcription factors and cofactors to access DNA-binding sites, thereby activating gene transcription. Specifically, acetylation of lysine 27 on histone H3 (H3K27ac) is strongly associated with active chromatin regions, including promoters and enhancers (9). In mouse GCs, growth differentiation factor 9 and bone morphogenetic protein 15 cooperatively recruit the histone acetyltransferase (HAT) p300 to the anti-Müllerian hormone promoter region. This recruitment is mediated through the PI3K–Akt and Smad2/3 pathways, leading to increased H3K27ac deposition and enhanced anti-Müllerian hormone expression (10). Conversely, in preovulatory follicular GCs, histone deacetylase 2 removes histone acetylation, leading to chromatin deactivation, reduced gene transcription, delayed cumulus expansion, and ovulation defects (11).

Similar to mice, the formation of PF in chickens occurs between day 1 and around day 5 post-hatch (12, 13), but the regulation of chromatin activation and repression in prefollicular GCs during this process is unknown. Furthermore, it is unclear to what extent changes in chromatin states influence the number of PFs that ultimately form. How does the newly formed PF quickly enter into quiescence in the fast-developing ovary. To address these questions, we isolated pre-GCs from chicken ovaries during the PF formation and conducted RNA-Seq and H3K27ac Cleavage Under Targets and Tagmentation (CUT&Tag) analysis. Our findings revealed a global reduction in chromatin activity, accompanied by decreased expression of associated genes in the pre-GCs during PF formation. Notably, enforced chromatin activation by inhibiting histone deacetylation in the *ex vivo*–cultured ovaries inhibited PF formation and led to unreleased germ cell cysts. These results underscore the importance of chromatin deactivation in the pre-GCs for successful PF formation and provide new insights into strategies for enhancing PF pool and ovarian reserves in chickens.

## Results

### The transcriptome analysis of pre-GCs during chicken PF formation

**Figure 1:**
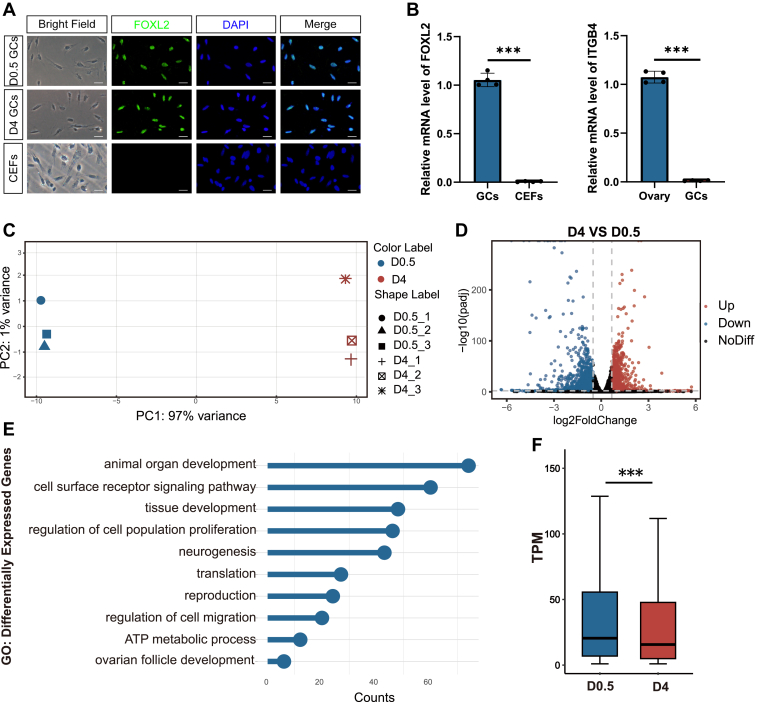
**Decreased transcriptionlevels in pre-granulosa cells (pre-GCs) during chicken primordial follicle establishment.***A*, immunofluorescence staining showed that the GC marker gene Foxl2 was expressed in isolated chicken pre-GCs, whereas chicken embryonic fibroblasts were used as a negative control. Scale bars represent 20 μm. *B*, RT–qPCR confirmed positive *Foxl2* expression (*left panel*) and no expression of the epithelial cell marker *ITGB4* (*right panel*) on isolated chicken pre-GCs. Data were mean ± SEM, n = 4, ∗∗∗*p* < 0.001 by Student's *t* test. *C*, principal component analysis (PCA) of RNA-Seq assay of D4 and D0.5 pre-GCs. *D*, scatter plot showing 530 genes significantly upregulated and 520 genes significantly downregulated at D4. |Fold change| ≥1.5, *p* < 0.05. *E*, Gene Ontology (GO) analysis of all differentially expressed genes showing the most enriched biological processes related to transcriptome changes. *F*, box plot showing significantly decreased mRNA levels of all expressed genes (TPM >1) in pre-GCs at D4 compared with D0.5. The median values of gene expression are 20.5119 TPM in D0.5 and 15.6932 TPM in D4. Data were mean ± SEM, ∗∗∗*p* < 0.001 by binomial test. Foxl2, Forkhead Box L2; ITGB4, integrin subunit beta 4; qPCR, quantitative PCR.

We first isolated pre-GCs from the D0.5 and D4 ovaries and verified the cell identity and purity. Immunofluorescence staining showed that Foxl2 (Forkhead Box L2), a pre-GC marker, was unanimously expressed in these cells, whereas the embryonic fibroblasts were Foxl2 negative (Fig. 1*A*). RT–quantitative PCR (qPCR) further showed high expression of *Foxl2* and low expression of *ITGB4* (integrin subunit beta 4) in the isolated cells (Fig. 1*B*), confirming that we had successfully obtained homogenous pre-GCs.

To identify the genes involved in PF establishment, we compared differentially expressed genes (DEGs) in pre-GCs before and after PF formation at D0.5 and D4, respectively. RNA-Seq revealed clear separation between the two stages in principal component analysis (PCA) (Fig. 1*C*). We identified 520 genes significantly downregulated and 530 genes upregulated during PF formation from D0.5 to D4, based on |fold change| ≥1.5 and *p* < 0.05 (Fig. 1*D*). Gene Ontology (GO) showed that the DEGs were involved in biological processes, such as "animal organ development," "cell surface receptor signaling," "neurogenesis," and "ATP metabolism" (Fig. 1*E*), which are related to ovarian tissue development and cell–cell communications during follicle formation, as well as metabolic regulations. In addition, we found that the mRNA levels of all expressed genes significantly decreased at D4 (Fig. 1*F*), indicating a reduction of an overall transcriptional activity in GCs during the PF formation, which could contribute to the establishment of a quiescent state.

### Reduced H3K27ac deposition and chromatin deactivation in the pre-GCs during PF establishment

**Figure 2:**
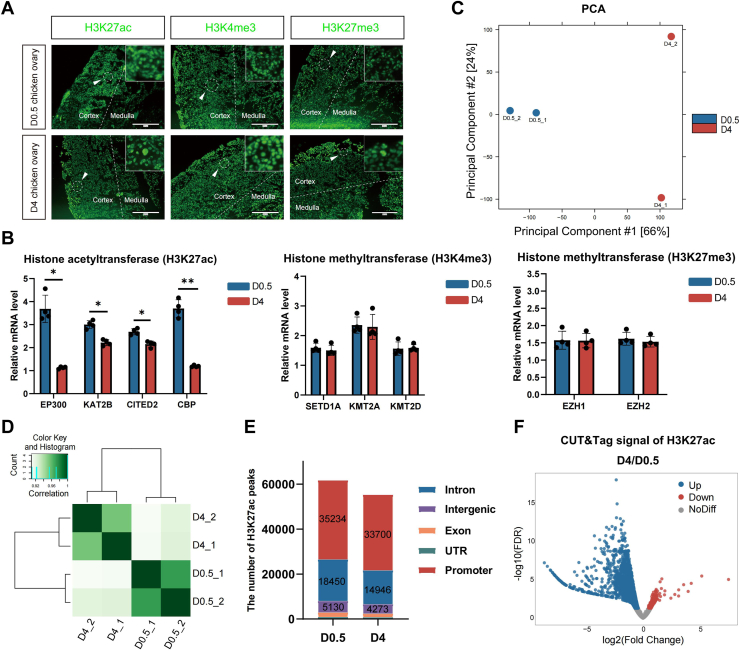
**Decreased deposition of H3K27ac during primordial follicle establishment.***A*, immunofluorescence staining of three histone modification markers, H3K27ac, H3K4me3, H3K27me3 on frozen sections of D0.5 and D4 chicken ovaries. Scale bars represent 50 μm. *B*, RT–qPCR showing significant downregulation of H3K27ac-related histone acetyltransferase enzymes during the process of primordial follicle establishment. The expression of H3K4me3 and H3K27me3-related histone methyltransferase enzymes was not changed. Data were mean ± SEM, n = 4, ∗∗*p* < 0.01, ∗*p* < 0.05 by Student's *t* test. *C*, principal component analysis (PCA) of the H3K27ac CUT&Tag assay of D4 and D0.5 pregranulosa cells. *D*, correlation analysis between biological replicates of CUT&Tag data. *E*, annotation of H3K27ac peak distribution on the genome at different time points. *F*, Volcano plot showing the differentially regulated H3K27ac peaks between D4 and D0.5 pregranulosa cells. CUT&Tag, Cleavage Under Targets and Tagmentation; D0.5, 0.5 days after hatching; D4, 4 days after hatching; H3K27ac, acetylation of lysine 27 on histone H3; H3K27me3, trimethylation of histone H3 lysine residue at site 27; H3K4me3, trimethylation of histone H3 lysine residue at site 4; qPCR, quantitative PCR.

To determine if the decrease in overall transcription levels is linked to changes in chromatin activity, we conducted immunofluorescence staining for three classical histone modification markers representing the chromatin activation or repression (H3K27ac, H3K4me3, and H3K27me3) on tissue sections of chicken ovaries at days D0.5 and D4. Our results revealed a significant reduction in the H3K27ac depositions in ovarian cells at D4 compared with D0.5, particularly in the cortex region where most follicles were located. In contrast, the levels of H3K4me3 and H3K27me3 histone markers remained largely unchanged (Fig. 2*A*). RT–qPCR further confirmed a significant decrease in the expression levels of histone acetylation modification enzymes, such as HAT Creb binding protein (*CBP*)/*EP300* (E1A binding protein p300) and *KAT2B* (lysine acetyltransferase 2b) (Fig. 2*B*). In contrast, the expression of histone methyltransferase genes associated with H3K4me3 and H3K27me3 remains unchanged (Fig. 2*B*). These findings suggest that the reduced transcriptional activity observed in pre-GCs during PF formation may be attributed to the decreased chromatin activity via histone deacetylation.

To explore the global H3K27ac deposition patterns and determine whether the decrease in chromatin activation caused the gene expression downregulation, we analyzed genome-wide H3K27ac-binding sites in pre-GCs at days D0.5 and D4 using CUT&Tag analysis. PCA of the H3K27ac CUT&Tag data revealed a clear separation between the two time points (Fig. 2*C*). Heatmap analysis demonstrated a strong correlation (*r* >0.88) between biological replicates within the same group (Fig. 2*D*). Then, we combined the data of the same stage to call H3K27ac peaks. Examination of genome-wide peak distributions showed a decrease in H3K27ac depositions at D4 compared with D0.5. The reduced H3K27ac peaks were predominantly located in intronic, promoter, and intergenic regions, affecting both proximal and distal regions from the transcription start sites (TSSs) of the genes (Fig. 2*E*). Furthermore, analysis of the strength of all H3K27ac depositions revealed a decrease in signal intensity at 15,791 peaks and an increase at only 338 peaks at D4 compared with D0.5 (Fig. 2*F*). These results indicate a clear downward trend in H3K27ac levels during the establishment of PFs, suggesting a shift toward chromatin deactivation in pre-GCs and the entry into cellular quiescence.

### Chromatin deactivation leads to downregulation of genes associated with cell apoptosis

**Figure 3:**
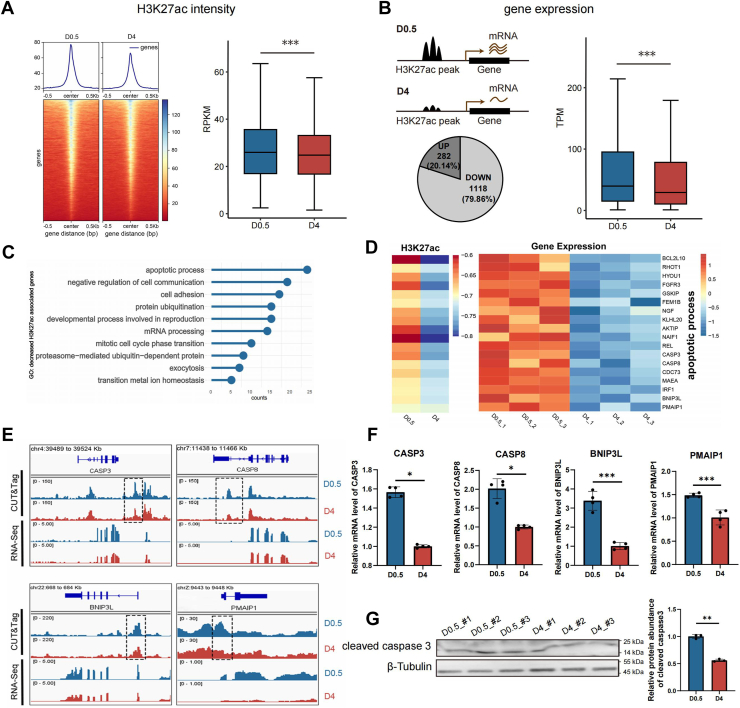
**Chromatin deactivation leads to the downregulation of genes associated with cell apoptosis.***A*, heatmaps (*left*) and boxplots (*right*) showing reduced H3K27ac levels at transcription start site (TSS) regions of all genes in D4 pregranulosa cells compared with D0.5. Data are presented as mean ± SEM, ∗∗∗*p* < 0.001 by binomial test. *B*, schematic illustration depicting how the loss of H3K27ac levels leads to decreased transcriptional activity of nearby genes. The pie chart indicates the proportion of genes associated with H3K27ac-depleted promoters that were either downregulated or upregulated from D0.5 to D4 (*left*). The box plot demonstrates that the mRNA expression levels were significantly reduced in the genes with decreased H3K27ac depositions within the TSS ±5 kb range (*right*). Data are presented as mean ± SEM, ∗∗∗*p* < 0.001 by binomial test. *C*, Gene Ontology (GO) analysis of genes with H3K27ac-depleted promoters, highlighting the most enriched biological processes such as apoptosis and negative regulation of cell communication. *D*, heatmap displaying the H3K27ac modification status in D4 pregranulosa cells (pre-GCs) and the mRNA expression levels of representative genes from the most enriched biological processes associated with the decrease in H3K27ac. *E*, IGV tracks showing gene expression and the H3K27ac CUT&Tag signal around the representative genes selected from *D*. *F*, RT–qPCR showing the expression levels of selected genes (from *D*) in D0.5 pre-GCs (*blue*) and D4 pre-GCs (*red*). Data were mean ± SEM, n = 4, ∗∗∗*p* < 0.001 by Student's *t* test. *G*, Western blot analysis of cleaved caspase-3 in pre-GCs from D0.5 and D4. CUT&Tag, Cleavage Under Targets and Tagmentation; D0.5, 0.5 days after hatching; D4, 4 days after hatching; H3K27ac, acetylation of lysine 27 on histone H3; IGV, Integrative Genomics Viewer; qPCR, quantitative PCR; TPM, transcripts per kilobase million.

To assess how the decreased H3K27ac deposition regulates the function of chicken pre-GCs during PF formation, we screened the genes potentially influenced by chromatin deactivation. First, we analyzed the dynamic changes of the H3K27ac signal within the proximal regulatory regions of genes, as the promoter activity directly influences gene transcription. Compared with D0.5, H3K27ac depositions at the TSS regions were greatly reduced on day D4 (Fig. 3*A*). We then examined the relationship between H3K27ac levels and the mRNA expression of adjacent genes to determine if H3K27ac-depleted gene promoters could lead to gene downregulation during PF establishment. Indeed, our findings indicate that gene expression levels positively correlate with H3K27ac modification intensity at gene promoters. Specifically, 1400 genes showed decreased H3K27ac depositions at their promoters, of which 1118 genes (79.86%) exhibited reduced mRNA expression. As a result, the overall transcription levels of the 1400 genes were significantly reduced (Fig. 3*B*).

Next, GO analysis of the genes with the proximal H3K27ac-depleted peaks showed that the most enriched pathways were related to "cell apoptosis" (Fig. 3*C*). This suggests that reduced chromatin acetylation could specifically target the cellular apoptosis in pre-GCs during PF establishment. Indeed, the heatmaps revealed a decrease in both H3K27ac signal levels and mRNA expressions of genes associated with cell apoptosis processes at D4 (Fig. 3*D*). The IGV (Integrative Genomics Viewer) of representative proapoptotic genes showed decreased H3K27ac CUT&Tag signals around the gene regulatory regions in D4 pre-GCs (Fig. 3*E*). For example, caspase-3 and caspase-8 are important enzymes in cell apoptosis (14), and we observed a decrease in H3K27ac signal in the promoter regions of *CASP3* and *CASP8*, leading to a significant reduction in their mRNA levels. The RT–qPCR confirmed their reduced expression levels at D4 (Fig. 3*F*). Furthermore, Western blot analysis of the cleaved caspase-3 indicated that the cellular apoptosis was greatly reduced during PF formation (Fig. 3*G*). In summary, the substantial reduction in H3K27ac deposition at proximal regulatory regions of cell apoptosis–related genes could contribute to alleviating cell death during PF establishment.

### Reduced enhancer activity impacts gene regulation and cell communication

**Figure 4:**
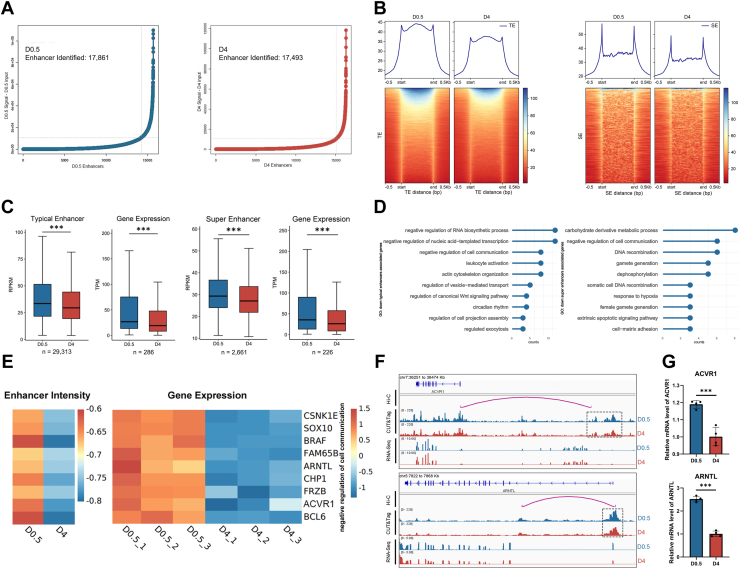
**Decreased enhancer activity leads to the repression of genes blocking cell communication.***A*, the enhancers were identified based on H3K27ac signals by using the ROSE software. A total of 17,861 and 17,493 enhancers were identified at D0.5 and D4, respectively. *B*, overall H3K27ac signals in D4 pregranulosa cells (pre-GCs) significantly decreased in both typical enhancers (D0.5: 15,704; D4: 16,270) and super enhancers (D0.5: 2,157; D4: 1,223). *C*, box plots showing significant decrease in H3K27ac signal intensity at both the typical enhancers and super enhancers in D4 compared with D0.5. Note that all the enhancers from both D0.5 and D4 cells were combined for analysis. The target genes connected with the weakened enhancers *via* chromatin interactions also showed significant decreased mRNA expression. Data were presented as mean ± SEM, ∗∗∗*p* < 0.001 by binomial test. *D*, Gene Ontology (GO) analysis of downregulated H3K27ac-related genes, showing the most enriched biological processes associated with the decrease in H3K27ac signals. *E*, heatmap displaying the reduced H3K27ac modification status in D4 pre-GCs and the mRNA expression levels of representative genes from the most enriched biological processes associated with the decrease in H3K27ac at enhancers. *F*, IGV tracks showing gene expression and the H3K27ac CUT&Tag signal around the representative genes and enhancers. *G*, RT–qPCR showing the expression levels of selected genes (from *E*) in D0.5 pre-GCs (*blue*) and D4 pre-GCs (*red*). Data were mean ± SEM, n = 4, ∗∗∗*p* < 0.001 by Student's *t* test. CUT&Tag, Cleavage Under Targets and Tagmentation; D0.5, 0.5 days after hatching; D4, 4 days after hatching; H3K27ac, acetylation of lysine 27 on histone H3; IGV, Integrative Genomics Viewer; qPCR, quantitative PCR.

To investigate how decreased H3K27ac depositions at the distal genomic regions influence gene transcription during PF establishment in chickens, we began by identifying potential enhancers regulated by chromatin acetylation. Using ROSE software, we identified 17,861 enhancers in D0.5 and 17,493 enhancers in D4, respectively (Fig. 4*A*). By integrating the previously identified chromatin interaction data in chicken GCs (15), we linked these enhancers to their corresponding target genes and examined gene expression levels.

First, we compared H3K27ac signal intensities in both typical enhancers and super enhancers (SEs) between D0.5 and D4. This comparison revealed a marked reduction in H3K27ac signal levels at D4 (Fig. 4*B*), indicating that the activity of both typical and SEs was dramatically decreased. Next, to assess the functional consequences of this reduction, we analyzed the correlation between the enhancer activity and the mRNA expression of associated target genes via chromatin interactions. Our findings showed that decreased H3K27ac signals on enhancers at D4 were accompanied by reduced transcription levels of their target genes (Fig. 4*C*). GO analysis of the weakened enhancer-targeted genes indicated that the "negative regulation of cell communication" pathway is most enriched (Fig. 4*D*). It suggests that diminished enhancer activity leads to the downregulation of negative regulators of cell communication. Heatmap analysis further confirmed that both H3K27ac signal levels and expression of representative genes involved in the negative regulation of cell communication were reduced (Fig. 4*E*).

Representative IGV tracks showed reduced H3K27ac CUT&Tag signals at distal enhancers, which linked to the target genes *via* chromatin interactions (Fig. 4*F*). Several of these genes, including ACVR1 (activin receptor type 1) (16), FAM65B (17), and FRZB (18), encode membrane-associated proteins implicated in blocking cell–cell communications. For instance, the SE target gene *ACVR1* showed decreased distal enhancer activities and reduced mRNA levels in D4. ACVR1 can block activin A signaling through forming a nonsignaling complex, thereby sequestering activin A and preventing signal transduction (16). The reduction of *ACVR1* at D4 could release this inhibition and enhance the intercellular communication in GCs through SMAD-dependent signaling (19). Therefore, we propose that decreased enhancer activity caused downregulation of *ACVR1* and other negative regulators, thereby promoting cell–cell communications and interactions in pre-GCs to facilitate PF assembly. RT–qPCR further confirmed the decreased expression of these selected target genes at D4 (Fig. 4*G*). Collectively, the reduction in the enhancer activity could downregulate the negative regulators of cell communication and thus facilitate cell-to-cell interactions to assemble the PF.

### Chromatin deactivation is necessary for PF formation in an *ex vivo* ovary culture model

To verify whether the H3K27ac reduction and the associated chromatin deactivation in pre-GCs is necessary for the establishment of PFs, we developed an *ex vivo* ovarian culture model and then manipulated the chromatin activation levels through altering the chromatin histone acetylation modification process.

**Figure 5:**
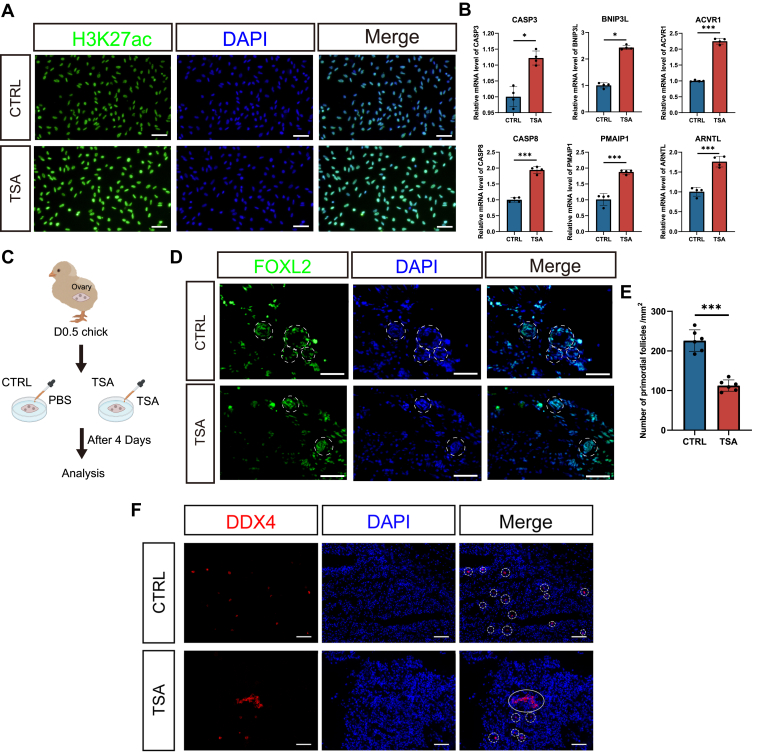
**Chromatin deactivation is necessary for the primordial follicle formation in *ex vivo* ovary culture.***A*, treatment of D0.5 pre-granulosa cells (pre-GCs) with TSA for 12 h resulted in an increase in H3K27ac levels. *B*, RT–qPCR analysis shows upregulation of selected genes (from Figs. 3*D* and 4*E*) after 24 h of TSA treatment of D0.5 pre-GCs. Data were mean ± SEM, n = 4, ∗∗∗*p* < 0.001, ∗*p* < 0.05 by Student's *t* test. *C*, schematic design of the *ex vivo* ovary culture experiment. *D*, representative Foxl2 immunofluorescence staining showed that the PFs were reduced after a 4-day TSA treatment of D0.5 ovarian tissue *in vitro*, as indicated by FOXL2-positive pre-GCs. *Dashed circles* represent primordial follicles. Scale bar represents 15 μm. *E*, bar graph demonstrates a significant decrease in the number of primordial follicles when ovaries are cultured with 1 μM TSA. Data represent mean ± SEM, n = 6, ∗*p* < 0.05 by Student's *t* test. *F*, after 4 days of TSA treatment of D0.5 ovaries *in vitro*, DDX4-positive oocytes showed that unruptured germ cell nests were increased. *Solid circles* indicate germ cell nests, and *dashed circles* indicate the single oocytes in the primordial follicles. Scale bars represents 50 μm. D0.5, 0.5 days after hatching; *Foxl2*, Forkhead Box L2; H3K27ac, acetylation of lysine 27 on histone H3; PF, primordial follicle; qPCR, quantitative PCR; TSA, trichostatin A.

Trichostatin A (TSA) is a potent and reversible HDAC inhibitor that prevents the removal of acetyl groups from histone lysine residues, thereby sustaining chromatin activation. TSA was widely used to promote gene transcription by maintaining chromatin histone H3K27 acetylation (20). We first treated D0.5 pre-GCs with TSA for 12 h and 24 h for testing the histone acetylation efficiency. The immunofluorescence staining showed increased H3K27ac levels upon TSA treatment (Fig. 5*A*), and the RT–qPCR confirmed the upregulation of representative target genes (Fig. 5*B*), indicating that the TSA-induced H3K27ac retention indeed maintained chromatin activation and increased target gene expression in pre-GCs. We then cultured D0.5 chicken ovaries *ex vivo* with TSA supplementation for 4 days (Fig. 5*C*). TSA treatment significantly enhanced H3K27ac deposition in ovarian cells during culturation, confirming increased chromatin acetylation (Fig. S1). Then, we employed the FOXL2 immunostaining to visualize the pre-GC distributions and identify the newly assembled follicles (Fig. S2). We observed a significant reduction in the number of assembled PFs in the ovary upon TSA treatment (Fig. 5, *D* and *E*). Therefore, the retention of H3K27ac and sustained chromatin activation resulted in a reduction in the establishment of PFs. We also found that the unreleased germ cell nests were increased (Fig. 5*F*), indicating the insufficient enclosure of germ cells with pre-GCs, which could be attributed to the compromised cell–cell interactions.

**Figure 6:**
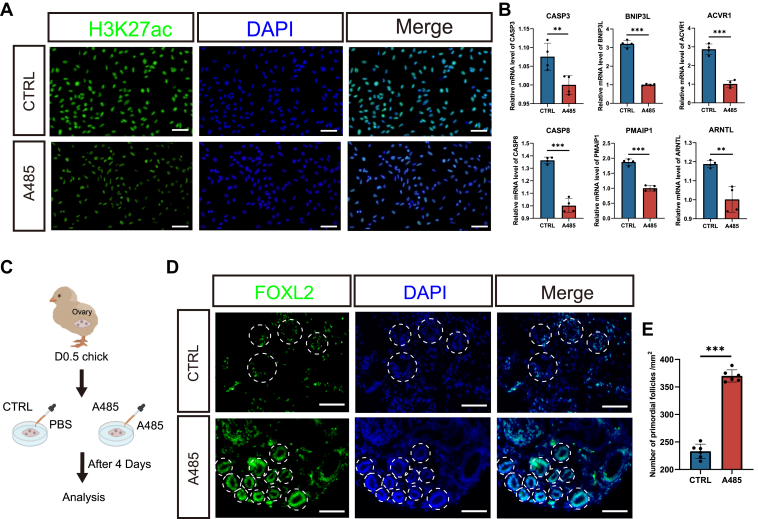
**Forcibly inactivate the chromatin could enhance the PF formation.***A*, treatment of pregranulosa cells at D0.5 with A-485 for 12 h leads to a decrease in H3K27ac levels. *B*, RT–qPCR analysis shows downregulation of selected genes (from Figs. 3*D* and 4*F*) after 24 h of A-485 treatment of D0.5 pregranulosa cells. Data were mean ± SEM, n = 4, ∗∗∗*p* < 0.001, ∗∗*p* < 0.01 by Student's *t* test. *C*, schematic design of the *ex vivo* ovary culture experiment. *D*, after 4 days of *in vitro* culture of D0.5 ovaries with A-485, FOXL2-positive staining show a significant increase in the number of established primordial follicles. *Dashed circles* indicate primordial follicles. Scale bars represent 15 μm. *E*, bar graph displays a significant increase in the number of primordial follicles when ovaries are cultured with 50 μM A-485. Data represent mean ± SEM, n = 6, ∗*p* < 0.05 by Student's *t* test. D0.5, 0.5 days after hatching; FOXL2, Forkhead Box L2; H3K27ac, acetylation of lysine 27 on histone H3; PF, primordial follicle; qPCR, quantitative PCR.

On the other hand, we tested whether enforced chromatin deactivation could enhance the PF formation. In this case, we used the HAT inhibitor A485 to block the histone acetylation and thus repress the chromatin activation (21). The A485 treatment could potently reduce the H3K27ac deposition and gene expression in chicken pre-GCs (Fig. 6, *A* and *B*). Upon supplementation of A485 during the *ex vivo* culturation of D0.5 ovaries (Fig. 6*C*), we found a significant increase in the number of PFs assembled compared with the control (Fig. 6, *D* and *E*). Thus, we collectively confirmed that chromatin deactivation is essential for the formation of PFs in chicken.

## Discussion

**Figure 7:**
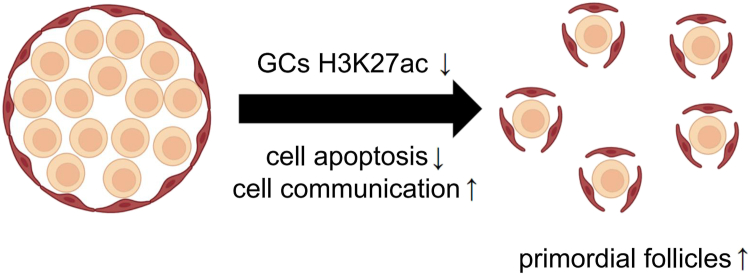
**Primordial follicle establishment in chicken was regulated by chromatin deactivation**.

The formation of PFs is the initial and critical step of animal folliculogenesis, laying the foundation for future follicle selection, ovulation, and overall reproductive performance. Recent studies have highlighted several critical cellular processes involved in PF formation, including the sufficient presence of pre-GCs (22, 23, 24), anti-apoptotic protection mechanisms (25, 26), and effective cell-to-cell communications (27, 28). However, the epigenetic mechanisms that control these cellular pathways remain to be discovered. Understanding the dynamic changes and intrinsic regulatory mechanisms in pre-GCs is essential for improving PF formation and enhancing ovarian reserve. In this study, pre-GCs were isolated from 0.5- and 4-day-old chickens, representing the onset and conclusion of the PF establishment process, respectively. By profiling gene expression and H3K27ac histone modifications in these cells, we provide evidence that modulation of chromatin activity is a key epigenetic mechanism governing PF assembly in chickens. Specifically, we identify a critical role of chromatin deactivation in regulating *de novo* follicle formation as well as the establishment and maintenance of their quiescent state (Fig. 7).

The proper assembly of PFs depends on the health and functionality of pre-GCs. However, the origin and development of pre-GCs remain controversial across species, with research progress hindered by the lack of specific molecular markers and reliable experimental models. A previous study suggested that only FOXL2-positive pre-GCs can encapsulate oocytes to form PFs (29). Moreover, LGR5-positive progenitor cells in the ovarian surface epithelium are considered the primary source of pre-GCs (7, 8, 23, ). In particular, these LGR5-positive progenitors proliferate and migrate inward during germ cell cyst breakdown, eventually differentiating into FOXL2-positive pre-GCs that envelop oocytes and form PFs (30). In the current chicken study, we confirmed the identity and purity of pre-GCs by FOXL2-positive staining, which allowed us to search for the factors regulating cellular function. Previous research suggested that several transcription factors, including SP1, bind to chromatin to regulate the proliferation of LGR5-positive cells, thus controlling the recruitment of pre-GCs and the maintenance of FOXL2-positive pre-GCs (31). These findings suggest that intrinsic transcription factors and chromatin regulations of pre-GCs may play crucial roles in the pre-GC differentiation and subsequent PF formation.

In the follicle, while most studies focus on epigenetic changes in oocytes, little is known about epigenetic regulations in pre-GCs (32, 33). Our study revealed a significant reduction in H3K27ac histone modification levels in pre-GCs during the formation of PFs, affecting the activity of both proximal gene promoters and distal enhancers. A concurrent decrease in H3K27ac-associated HATs further supports the overall depletion of this active chromatin mark. Furthermore, the chromatin deactivation caused an overall reduction of transcriptional activity to facilitate quiescence entry as well as counteract apoptosis in the PFs. Since approximately two-thirds of oocytes are eliminated by programmed cell death during the formation of PFs, both apoptosis and autophagy are central to this process (25, 26). We speculate that a large number of pre-GCs may die along with the death of oocytes during follicle formation. However, in the GO analysis of downregulated genes near the depleted H3K27ac peaks within the gene proximal region, we found that “cell apoptosis” was most enriched among the reduced biological activities. This supports the idea that reduced apoptotic activity enables more pre-GCs to survive and interact with oocytes. This is further evidenced by the downregulation of classical apoptosis-related genes, *Caspase3* and *Caspase8*, as well as the cleaved-caspase-3 protein. Therefore, it appears that pre-GCs may utilize an intrinsic antiapoptotic protective strategy to counteract the death signals from the germ cells to establish a follicle. Consequently, only oocytes successfully encapsulated by surviving pre-GCs persist. In addition, GO analysis of downregulated genes regulated by distal enhancers associated with reduced H3K27ac were enriched in “negative regulation of cell communication.” This aligns with the observed downregulation of ACVR1 (a bone morphogenetic protein type I receptor), which inhibits transforming growth factor β–activin pathway signaling, a key regulator in granulosa cell interaction and function (34, 35). These findings indicate enhanced intercellular communications in pre-GCs as well as between pre-GCs and germ cells following the downregulation of H3K27ac. The strengthened germ–somatic cell interactions are required for the efficient assembly of a follicle structure.

To examine the functional role of H3K27ac during PF formation, we conducted chromatin modulation experiments using the HAT activator TSA and the HDAC inhibitor A-485. Modulating H3K27ac levels changed the expression of target genes involved in apoptosis and cell communication. Notably, reducing H3K27ac levels with A-485 in cultured ovaries led to an increased number of PFs, whereas TSA-mediated elevation of H3K27ac levels resulted in fewer PFs and delayed germ cell nest breakdown. These findings underscore the critical role of chromatin deactivation during PF formation. The chromatin deactivation in pre-GCs could prevent the germ cell apoptosis and promote intercellular interactions, the two key cellular features necessary for the successful assembly of a follicle. We hypothesized that, in the highly dynamic and fast-developing early ovary, this specific chromatin deactivation is temporary but necessary for PF formation. This deactivated chromatin state enables PFs to enter dormancy, allowing them to be preserved until reactivated during later stages of follicular development. Eventually, GCs must resume activity to support follicle growth to produce eggs in the future.

## Conclusion

The current study investigated gene expression and chromatin regulation in chicken pre-GCs during PF formation, revealing a global reduction in active chromatin, which in turn downregulated the genes promoting apoptosis and blocking cell–cell communications in order to facilitate follicle assembly and subsequent transition into a quiescent state. Extended chromatin activation through HDAC inhibitors significantly reduced PF numbers and preserved unruptured germ cell cysts, highlighting the chromatin manipulations that could improve PF establishment. This study offers new insights into the epigenetic mechanisms that regulate follicle formation and provide strategies for improving reproductive reserves in laying hens and other animals.

## Experimental procedures

### Animal and tissue preparation

Newly hatched female Hy-Line Brown chicks, a widely used layer breed, were purchased from the local Dongyue Poultry Hatchery at the Shandong Agricultural University. The chicks were divided into two groups and reared in chicken cages at 35 °C for either 0.5 or 4 days (D0.5 or D4) before being euthanized. All procedures are approved by the Shandong Agricultural University Animal Care and Use Committee, in accordance with the principles outlined in the Guide for the Care and Use of Laboratory Animals (permit number: SDAUA-2022-95).

### Isolation of pre-GCs

The pre-GCs were isolated according to a previous protocol with slight modifications (36, 37). In brief, the ovaries from D0.5 or D4 Hy-Line Brown chicks were dissected and placed in sterile culture dishes. The tissue was washed two to three times with PBS and then cut into small fragments using sterile ophthalmic scissors. A digestion solution containing 0.25% trypsin and 0.04% EDTA was added to the tissue and incubated at 37 °C for 10 min. The digestion was halted by adding 10% fetal bovine serum (CELLiGENT; CG0430A). After centrifugation, the supernatant was removed, and the pellet was further digested with 2 mg/ml collagenase II (Solarbio; C8150) at 37 °C for 30 min. The digestion mixture was then filtered through a 40 μm strainer to remove oocytes. The resulting cell suspension was centrifuged and resuspended in GC culture medium (Dulbecco's modified Eagle's medium supplemented with 10% fetal bovine serum and 1% penicillin–streptomycin solution). The cells were counted, seeded in 24-well plates, and incubated at 37 °C with 5% CO_2_ overnight. After 10 to 12 h, the media were carefully replaced, and the GCs adhered to the base of the plate. The great majority of resulting cells were GCs (38), and according to the purpose of the assay, different numbers of ovaries were harvested for achieving enough cell yields. The cell treatment time was indicated in each figure legend. All steps were performed using aseptic techniques to ensure sterility and prevent contamination.

### Culture of ovaries

The *ex vivo* ovary culture protocol was adopted from previous studies with optimizations (39, 40). In brief, the ovaries from D0.5 Hy-Line Brown chicks were dissected and washed with PBS two to three times, followed by culture in a 24-well cell culture plate (NEST) placed in a 39 °C, 5% CO_2_ incubator. The basal culture medium consisted of Dulbecco's modified Eagle's medium (Gibco), 100 IU/ml penicillin, 100 μg/ml streptomycin, and 10% fetal bovine serum (CELLiGENT; CG0430A). The ovaries were treated with 50 μM A-485 (MCE; HY-107455) or 1 μM TSA (MCE; HY-15144) in the culture. A-485 is a potent and selective catalytic inhibitor of p300/CBP. TSA is an effective and specific inhibitor of HDAC types I and II (HDAC class I/II) (41). The inhibitor treatment time was indicated in each figure legend, and the ovaries were fixed in 4% paraformaldehyde for histological studies.

### Immunofluorescence staining

For cell immunofluorescence staining, the cell culture medium was discarded, and cells were washed with PBS. Cells were fixed in 4% paraformaldehyde at room temperature for 10 min and permeabilized with 0.5% Triton X-100 for 10 min. After blocking with 10% serum for 1 to 2 h, primary antibodies were applied: Anti-FOXL2 (1:200 dilution, BOSTER; A01185-1) and Anti-Histone H3 (acetyl K27) (1:200 dilution, Abcam; ab4729), followed by overnight incubation at 4 °C. Secondary antibodies, Alexa Fluor 488 donkey anti-rabbit IgG (1:200 dilution, Invitrogen; A-21206), were added and incubated at room temperature in the dark for 1 to 2 h. Cells were then stained with 4′,6-diamidino-2-phenylindole for 5 to 10 min before observation under a fluorescence microscope (42).

For frozen tissue sections, ovarian tissues were fixed in 4% paraformaldehyde overnight at 4 °C, dehydrated in 30% sucrose for 2 to 4 h, embedded in optimal cutting temperature compound, and rapidly frozen in liquid nitrogen. Sections of 5 μm thickness were prepared and fixed with 4% paraformaldehyde at room temperature for 10 min. After permeabilization with 0.5% Triton X-100, sections were blocked with 10% serum for 1 to 2 h. Primary antibodies were applied, including Anti-FOXL2 (1:200 dilution; BOSTER, A01185-1) (38), Anti-DDX4 (1:200 dilution; Abcam, ab13840) (43), TriMethyl-Histone H3-K27 (1:200 dilution, ABclonal; A2363), Anti-Histone H3 (acetyl K27) (1:200 dilution, Abcam; ab4729), and Anti-Histone H3 (trimethyl K4) (1:200 dilution, Abcam; ab8580). After overnight incubation at 4 °C, secondary antibodies (Alexa Fluor 488 donkey anti-rabbit IgG, 1:200 dilution, Invitrogen; A-21206) were added. The DAPI (4′,6-Diamidino-2-phenylindole) staining was followed, and sections were observed under a fluorescence microscope (44). For the calculation of GCs, we counted three fields of view from the same sample (ovary-derived cells) in a total of three biological samples (three batches of ovary-derived cells). For the calculation of follicles, three sections were counted and averaged for each sample (ovary) from a total of five biological samples (five ovaries). Germ cells (DDX4^+^) not surrounded by pre-GCs (FOXL2^+^) were scored as unassembled (remaining in nests). Oocytes surrounded by pre-GCs were scored as PFs.

### RNA extraction

Total RNA was extracted from ovarian pre-GCs at D0.5 and D4 using the RNAsimple Total RNA Kit (TIANGEN; DP419). RNA quantity and quality were assessed using a NanoDrop 2000 spectrophotometer (Thermo), and integrity was verified during analysis.

### Real-Time qPCR

Total RNA isolated from the ovarian pre-GCs of Hy-Line Brown chickens at D0.5 and D4 was used for RT–qPCR. The total RNA (500 ng) was reverse transcribed into single-stranded complementary DNA using an RT kit (Yugong Biolabs; EG15133S). Differential gene expression was analyzed using a reaction system (ACCURATE BIOTECHNOLOGY; AG11718) on the Applied Biosystems QuantStudio Q5. The relative expression levels of each gene were estimated using the 2-ΔΔCT method. Primers were designed using Primer 5.0 as shown in Table S1.

### Western blot

Cells were washed three times with ice-cold PBS. A mixture of Western/immunoprecipitation lysis buffer (NCM; P70100) and protease inhibitor cocktail (NCM; P001) at a 50:1 ratio was added to the cells, followed by lysis on ice for 5 min. Lysates were then centrifuged at 15,000 rpm for 5 min at 4 °C. Protein concentrations were measured using the BCA Protein Assay Kit (NCM; WB6501) following the manufacturer's instructions. Proteins were mixed with 5× loading buffer (Epizyme; LT101S), boiled at 100 °C for 12 min to ensure complete denaturation, and 30 μg of protein per sample was separated by SDS-PAGE and transferred onto a polyvinylidene fluoride membrane (Millipore; ISEQ00010). Membranes were blocked with rapid blocking buffer (NCM; P30500) for 20 min and incubated overnight at 4 °C with primary antibodies, including anti-Caspase-3 (Proteintech; 66470-2-Ig) and β-Tubulin (Abclonal; AC021). After washing with Tris-buffered saline with Tween-20, membranes were incubated with horseradish peroxidase–conjugated anti-mouse secondary antibody (Beyotime; A0208) for 2 h. Detection was performed using a high-sensitivity ECL substrate (NCM; P10100), and signals were visualized with a C300 imaging system.

### RNA-Seq data analysis

High-throughput RNA-Seq was performed using Illumina NovaSeq 6000. The RNA-Seq raw data were cleaned by Fastp, and the clean data were aligned to the reference chicken genome (Ensembl, GRCg6a) utilizing Hisat2. The gene read counts were calculated by featureCounts and normalized to the transcripts per kilobase million (TPM). Differential expression analysis was conducted using the R package DESeq2, applying criteria of |fold change| ≥1.5 and *p* < 0.05 to identify DEGs. GO analysis was executed with the clusterProfiler package. The R packages ggplot2 and pheatmap were employed to produce the graphical representation of plots of PCA and heatmap. Genes with TPM ≥1 at least in one sample were defined as detected genes.

### CUT&Tag assay

The operations were carried out using the Hyperactive Universal CUT&Tag Assay Kit for Illumina (Vazyme; TD901) according to previous protocol (45). Briefly, 1 × 10^6^ pre-GCs from D0.5 or D4 Hy-Line Brown chicken ovaries were washed with 500 μl of wash buffer and centrifuged at 1000 rpm for 5 min at room temperature. The cells were resuspended in 100 μl of wash buffer. Concanavalin A-coated magnetic beads (10 μl) were washed twice with 100 μl of binding buffer and added to the cell suspension, followed by incubation at room temperature for 10 min. After removal of the supernatant, the cells mixed with the beads were resuspended in 200 μl of antibody buffer containing 2 μg of Rabbit anti-H3K27ac antibody (abcam; ab4729) or Rabbit control IgG antibody (abclonal; AC005). The mixture was incubated at room temperature for 3 h, and the supernatant was removed using magnetic separation. Then, 1 μl of Goat anti-Rabbit IgG H&L (abcam; ab6702) diluted in 100 μl of Dig-wash buffer was added to the cells. After three gentle washes with 800 μl of Dig-wash buffer, 100 μl of 0.04 μM Hyperactive pG-Tn5 Transposon was added to the cells. The mixture was incubated at room temperature for 1 h, followed by three gentle washes with 800 μl of Dig-300 buffer. The pG-Tn5 was activated by adding tagmentation buffer containing Mg^2+^. After incubating at room temperature for 1 h, the reaction was terminated by adding 10 μl of 0.5 M EDTA, 3 μl of 10% SDS, and 2.5 μl of 20 mg/ml proteinase K. The samples were incubated overnight at 37 °C. DNA fragments were then isolated using phenol–chloroform, isopropanol, and GlycoBlue and dissolved in TE buffer. Subsequently, the DNA was amplified using N5 and N7 primers (Vazyme; TD202) and purified using a DNA purification kit (JianShi Biotech; TD413) for high-throughput sequencing. All CUT&Tag libraries were sequenced on an Illumina NovaSeq 6000 platform in PE150 mode by Nanjing Jiangbei Biomedical Platform Co, Ltd.

### CUT&Tag data analysis

The CUT&Tag raw data were evaluated by FastQC, and low-quality reads were cleaned using Fastp. The paired-end reads were aligned to the reference chicken genome (Ensembl, GRCg6a) using Burrows-Wheeler Aligner tool. Peak calling was executed with MACS2, employing parameters set at *p* = 0.05 and incorporating corresponding input controls. SAMtools was used to merge peaks from two replicates. HOMER was used for peak annotation with the “annotatePeaks” function. The region within ±5 kb of the TSSs was defined as the promoter, corresponding to the proximal regulatory region. Intergenic regions were classified as distal regulatory elements, whereas exons, UTRs, and introns were collectively referred to as the gene body. Alignment results were then converted to coverage bigwig files, and the bigwig formats can be visualized by the software IGV. DeepTools were applied for the normalization of CUT&Tag profiles, and data within ±5 kb around the TSS were quantified in fragments per kilobase million. The R package Diffbind was used for PCA, correlation analysis, and differential peak identification (|fold change| ≥1, false discovery rate <0.05). Gene expression around ±5 kb of the differential peak was calculated to perform correlation analysis between H3K27ac modification and gene expression patterns, only genes expressed at least in one sample (TPM ≥1) were included.

### Genes associated with enhancers

ROSE software was used to identify enhancers based on H3K27ac signal and ranked all the enhancers with default parameters. Neighboring enhancer elements (within 12.5 kb) were defined and merged based on H3K27ac signal and then ranked to identify an inflection point. Enhancers surpassing this inflection point were denoted as SE peaks, whereas those falling below were categorized as typical-enhancer peaks. The signal strength of enhancers was computed using DeepTools, with a threshold for differential enhancers set at |fold change| ≥1.1 (46). Leveraging available chromatin interaction loops information (15), we located the enhancer anchor, matching it with our enhancer peaks to identify the gene located at the opposite end of the chromatin loops. We identified target loops by overlapping our predicted enhancer positions with enhancer anchor sites, requiring that the enhancer start and end positions fall within the anchor coordinates. The target genes of the enhancers were found through these loops, and then the correlation analysis of the enhancers and gene expression patterns was carried out, finally the GO enrichment analysis of the enhancer-regulated genes was performed.

### Statistical analysis

The statistical tests used in this study were performed using R, v4.2.2 or SPSS, v26.0, and details of statistical analyses were described within the Experimental procedures section. Statistical analyses were executed using the independent-sample Student's *t* test (two-sided) to assess the obtained results. Furthermore, the exact binomial test was employed for the comparative analysis of gene expression within the ±5 kb vicinity of differential peaks. Experimental outcomes were succinctly presented as the mean ± SEM, with statistical significance denoted as follows: ∗*p* < 0.05, ∗∗*p* < 0.01, and ∗∗∗*p* < 0.001.

## Data availability

All raw RNA-Seq data and CUT-tag data generated in this study were deposited in the National Center for Biotechnology Information Sequence Read Archive under the accession number PRJNA1162732 and are available at https://dataview.ncbi.nlm.nih.gov/object/PRJNA1162732?reviewer=t1qbr91qelceg1nvhm7v9e41jh.

## Supporting information

This article contains supporting information.

## Ethics approval and consent to participate

Guidelines for utilization of fertilized eggs and animals in our study were followed according to the standards of Shandong Agricultural University Animal Care and Use Committee.

## Conflict of interest

The authors declare that they have no conflicts of interest with the contents of this article.
