# Supplementary material for: Chromatin deactivation in pregranulosa cells contributes to primordial follicle formation
Source: J Biol Chem. 2025 Aug 14;301(10):110598. doi: 10.1016/j.jbc.2025.110598 (PMC12466267; doi:10.1016/j.jbc.2025.110598)
Supplement: Supporting Figures S1–S3 and Table S1 [file mmc1.docx]

**Supplementary figure 1**

**
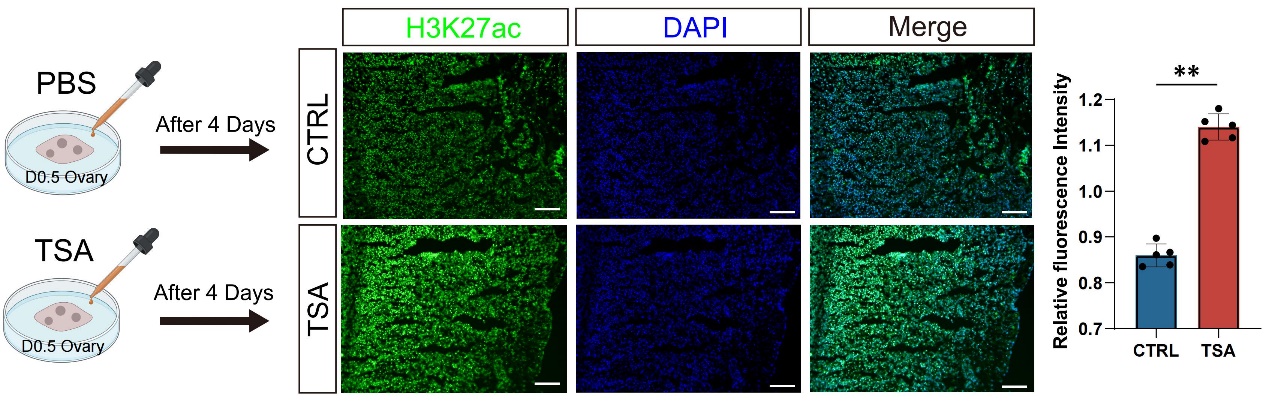
**

**Figure S1. The TSA treatment during ex vivo ovary culture increased chromatin acetylation.**

The ovaries were collected from D0.5 chicken and cultured ex vivo for 4 days. The TSA treatment significantly increased the chromatin histone acetylation (H3K27ac) in the ovary as shown by the H3K27ac immunostaining of tissue sections. Data represent mean ± SD. Data are analyzed by unpaired t test. **p < 0.01. Scale bar: 25 μm.

**Supplementary figure 2**

**
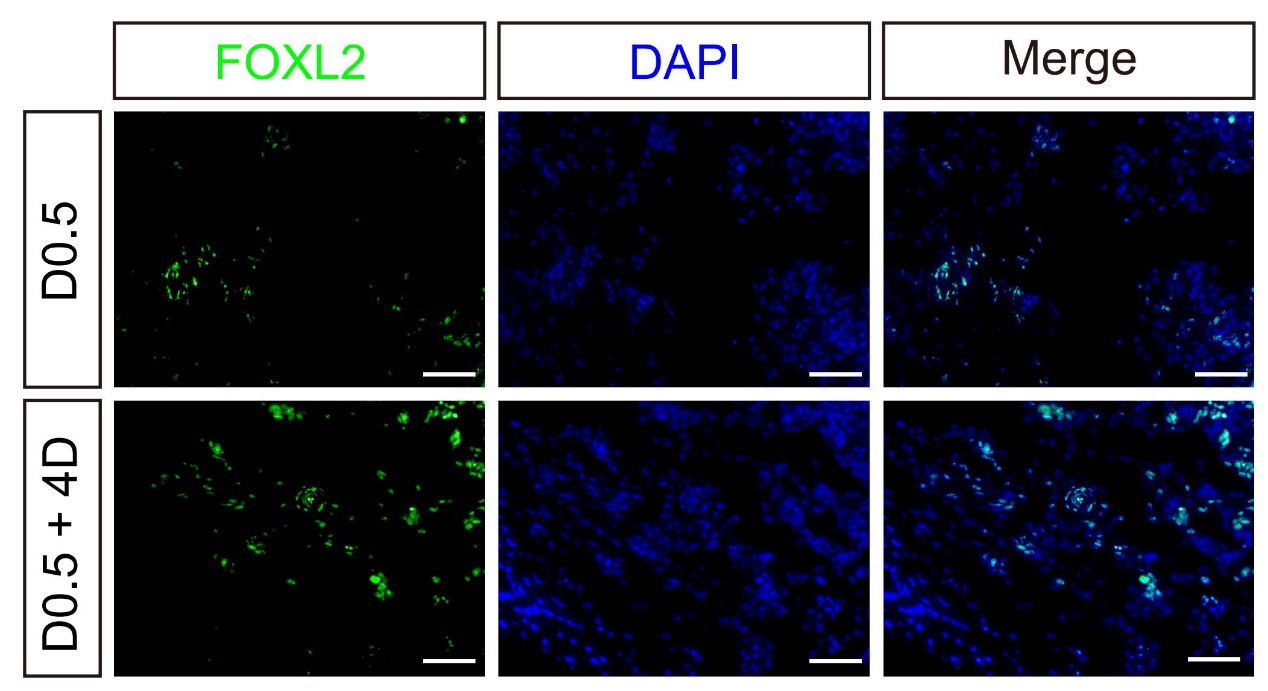
**

**Figure S2. Analysis of FOXL2-positive pre-granulosa cells during ex vivo culture of ovaries.**

The ovaries were collected from D0.5 chicken and cultured ex vivo for 4 days. FOXL2 immunostaining showed that FOXL2-positive pre-granulosa cells increased during the culture period. Scale bar: 20 μm.

**Supplementary figure 3**

**
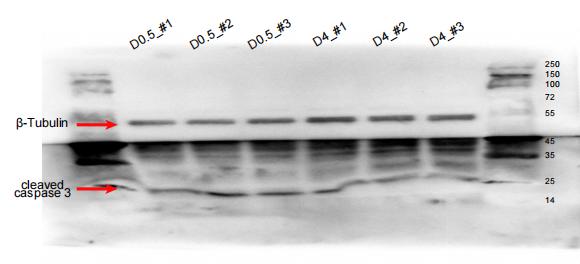
**

**Figure S3. The image of uncropped gel from Western blot.**

**Supplementary Table 1. Quantitative real-time PCR primer sequences used in this study**

| Gene | Gene ID | Primer Sequence |
| --- | --- | --- |
| FOXL2 | 503512 | Forward：TGTCCGGGATCTACCAGTAC  Reverse：CTCGAACATGTCCTCGCA |
| ITGB4 | 417374 | Forward：CAACATCAACTACTTCAGCC  Reverse：TGTCCTTCCTCCAGCCAATC |
| EP300 | 418000 | Forward：CCTAAAGCGGCAGAATCACC  Reverse：CATCAATCTCCTCAAAGGCAAA |
| KAT2B | 428441 | Forward：GAAGAACCCCAACCCACCTC  Reverse：CCCCAGCAGCCTATTCATTT |
| CITED2 | 395399 | Forward：CGGACAGCATTTCAGGGACT  Reverse：CCCATTTCGATGACTAAGGACA |
| CBP | 395099 | Forward：TCTCCAGCACTTCCAACCCA  Reverse：TTCTTCCTGCGACATTCACG |
| SETD1A | 416851 | Forward：CTCAGATAACGAGGTCCGAATG  Reverse：GAGTCAGGTAATGGCGTGGG |
| KMT2A | 414897 | Forward：TTAGGCATCGTGACCTCGGTA  Reverse：GGGTTTGGTTGGGTAGTTTGG |
| KMT2D | 425846 | Forward：CGGTCGTCCTCTTCTCCAAC  Reverse：CTTCAGCAGCATCACCTTCG |
| EZH1 | 420023 | Forward：AAATCGCCACCCCTCCAA  Reverse：GAGCTGCCGAAGACGCATA |
| EZH2 | 420784 | Forward：GCCCTGACTGCTGAGCGTAT  Reverse：GGTCTGCTGGTGTTGTTTGGA |
| CDH2 | 414745 | Forward：CCTACAGCCCCACCATACGA  Reverse：TCTTGCTCACCACCGCTACTT |
| CDH4 | 419222 | Forward：CTGCTCCACCACCACTCT  Reverse：TGCTGCTCGCTTACCTTCTA |
| CASP3 | 395476 | Forward：CTGAAGGCTCCTGGTTTA  Reverse：TGCCACTCTGCGATTTAC |
| CASP8 | 395284 | Forward：GGTGAGCAGCAAGATTGACA  Reverse：CTGCCTCTGCTCCCATTTAG |
| ARNTL | 374115 | Forward：GGCATACTTACCCCAGGAACTT  Reverse：TCATGAAACTGAACCAGCGACT |
| ACVR1 | 395246 | Forward：CTCATCGGGACTTGAAGAGCA  Reverse：CGGAGCCATGTAGCGTTTG |
| GAPDH | 374193 | Forward：GAGGGTAGTGAAGGCTGCTG  Reverse：CACAACACGGTTGCTGTATC |
